# Encapsulation and assessment of therapeutic cargo in engineered exosomes: a systematic review

**DOI:** 10.1186/s12951-023-02259-6

**Published:** 2024-01-03

**Authors:** Zhen Chen, Min Xiong, Jiaqi Tian, Dandan Song, Shuyin Duan, Lin Zhang

**Affiliations:** 1 Clinical Medical Research Center for Women and Children Diseases, Key Laboratory of Birth Regulation and Control Technology of National Health Commission of China, Shandong Provincial Maternal and Child Health Care Hospital Affiliated to Qingdao University, Jinan, 250001 China; 2 Key Laboratory of Birth Defect Prevention and Genetic Medicine of Shandong Health Commission, Jinan, 250001 China; 3 School of Public Health, Weifang Medical University, Weifang, 261000 China; 4 School of Public Health, North China University of Science and Technology, Tangshan, 063000 China; 5 School of Public Health, Shandong First Medical University & Shandong Academy of Medical Sciences, Jinan, 250001 China

**Keywords:** Engineered exosomes, Drug encapsulation, Efficacy assessment, Clinical translation

## Abstract

Exosomes are nanoscale extracellular vesicles secreted by cells and enclosed by a lipid bilayer membrane containing various biologically active cargoes such as proteins, lipids, and nucleic acids. Engineered exosomes generated through genetic modification of parent cells show promise as drug delivery vehicles, and they have been demonstrated to have great therapeutic potential for treating cancer, cardiovascular, neurological, and immune diseases, but systematic knowledge is lacking regarding optimization of drug loading and assessment of delivery efficacy. This review summarizes current approaches for engineering exosomes and evaluating their drug delivery effects, and current techniques for assessing exosome drug loading and release kinetics, cell targeting, biodistribution, pharmacokinetics, and therapeutic outcomes are critically examined. Additionally, this review synthesizes the latest applications of exosome engineering and drug delivery in clinical translation. The knowledge compiled in this review provides a framework for the rational design and rigorous assessment of exosomes as therapeutics. Continued advancement of robust characterization methods and reporting standards will accelerate the development of exosome engineering technologies and pave the way for clinical studies.

**Graphical Abstract:**

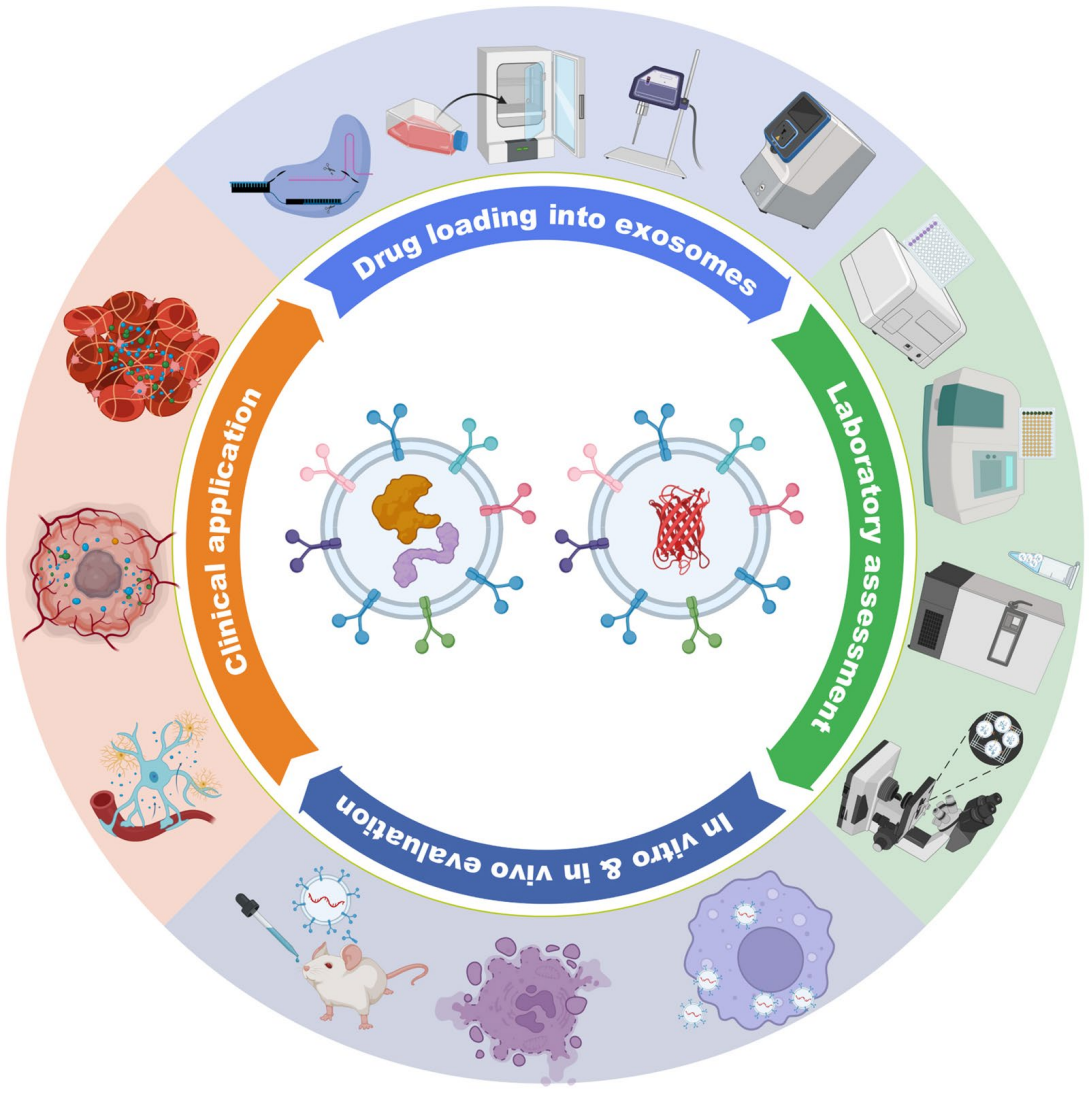

## Introduction

Exosomes are nanoscale extracellular vesicles secreted by cells that have recently emerged as promising drug delivery vehicles. Functionally, exosomes possess a lipid bilayer membrane that encloses bioactive cargoes including proteins, lipids, and nucleic acids. With high biocompatibility and low immunogenicity, exosomes can facilitate targeted drug transport with reduced immune clearance [1]. However, the clinical potential of natural exosomes is limited by their short circulation half-lives, variability based on cell source, and insufficient drug loading capacity. To overcome these limitations, researchers have developed engineered exosomes through bioengineering techniques to enhance drug encapsulation and targeting. Generally, engineered exosomes hold increased drug loading and stability compared to natural exosomes by preventing exosome aggregation, adsorption, and rapid clearance, and the engineering of exosomes to create tailored and efficient drug delivery systems has become an intense area of investigation across biomedical and biotechnology fields.

**Fig. 1:**
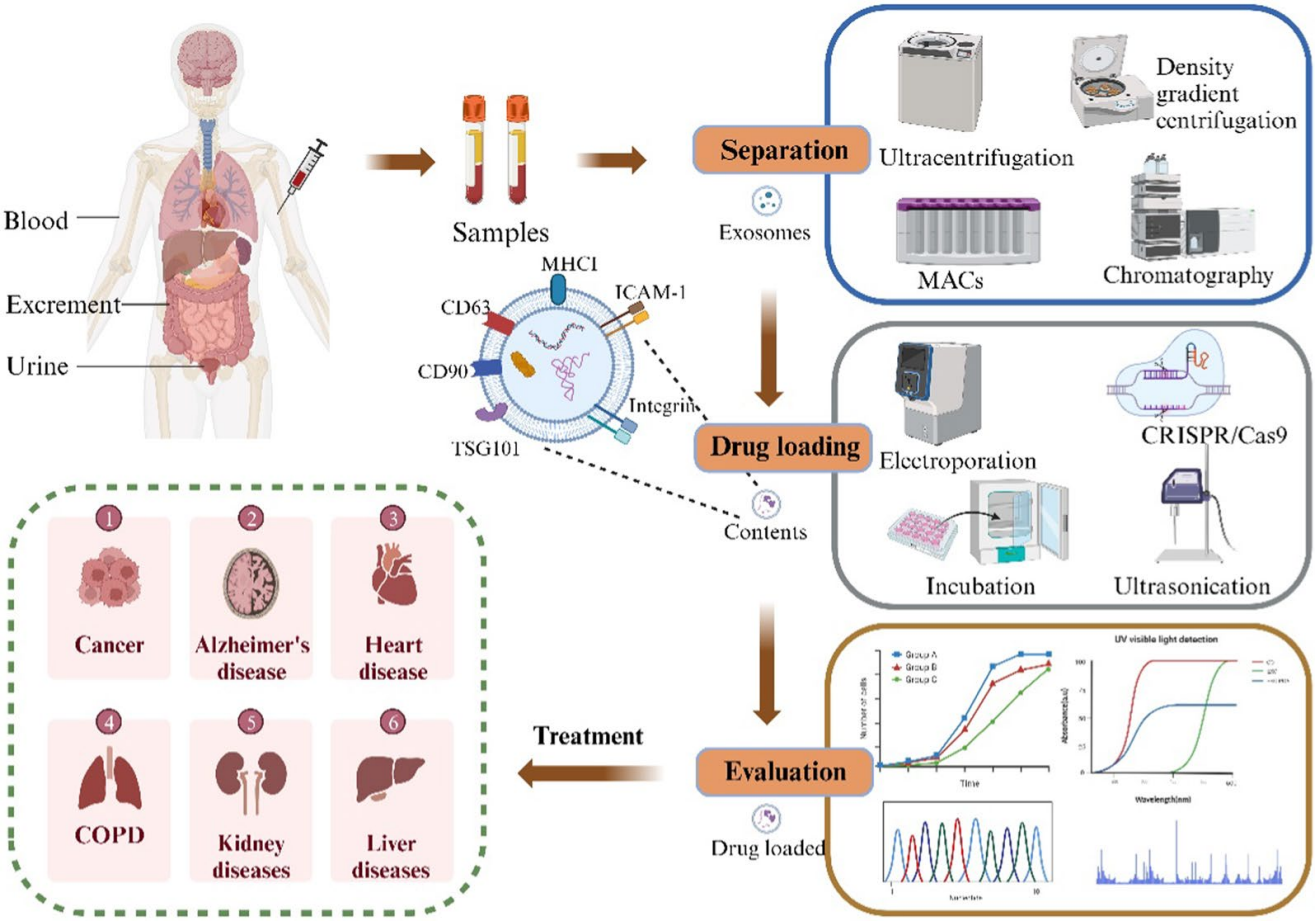
Overview of the process for engineering exosomes for therapeutic applications. Natural exosomes are first isolated from body fluids, then they are engineered to carry specific cargoes or express ligands on their surface. The drug loading capability of the engineered exosomes is evaluated before studying their potential clinical uses

Bioengineering techniques can enhance exosome production, stability, and purity, and engineered exosomes generated through genetic modification of parent cells have emerged as promising drug delivery vehicles (Fig. 1). Besides, these engineered exosomes offer numerous advantages, such as drug protection, increased stability and bioavailability, reduced toxicity, and improved targeting ability. As a result of these benefits, engineered exosomes are currently being explored as efficient drug carriers in the field of therapeutic delivery [2]. Although significant technical progress has been made in exosome engineering, yet demonstrations on the evaluation of exosome therapeutic efficacy, standardized methods for fabrication, drug loading, and quantification remain underdeveloped, and the lack of robust methods has hindered the clinical translation of engineered exosomes. Therefore, this review synthesizes recent progresses in isolation, drug encapsulation, and characterization of engineered exosomes with special focus on evaluating their drug delivery capacity. The establishment of reliable engineering guidelines and assessment strategies will facilitate the clinical development of exosome engineering technologies. At last, we outline current challenges and future directions in translating engineered exosomes from bench to bedside.

## Exosome isolation, purification, and drug encapsulation

### Isolation and purification of exosomes

The isolation and purification of exosomes involve a variety of techniques, each with its own set of advantages and limitations. These techniques include ultracentrifugation, filtration, differential centrifugation, density gradient centrifugation, immunomagnetic separation, and size exclusion chromatography (Table 1). The choice of method often results in a trade-off between exosome purity and yield [3]. For instance, while ultracentrifugation might yield a high volume of exosomes, it may not provide the same level of purity as immunomagnetic separation. Conversely, methods that offer high purity, such as size exclusion chromatography, might not yield as many exosomes. Therefore, the selection of an appropriate method depends on the specific requirements of the study or application, and it is crucial to consider both the advantages and limitations of each method to make an informed decision.

**Table 1:** Summary and comparison of different exosome isolation methods

Methods	Advantages	Disadvantages
Ultracentrifugation	Obtaining exosomes with high concentration suitable for large-scale isolation	Complex instrument operation and low efficiency of exosome isolation and extraction
Filtration	High exosome purity, short time expenditure, and ability to fractionate exosomes by size	Membrane pore size needs to be selected based on sample characteristics, and the filtration rate may be affected by pore size and sample concentration
Differential centrifugation	The extracted exosomes have high purity, which ensures the structural integrity of the exosomes	Multiple centrifugation steps required, resulting in cumbersome workflow
Density gradient centrifugation	Efficient isolation of exosomes of different densities	Preparing density gradient solutions requires technical expertise and experience. Centrifugation speed and duration need to be properly controlled, otherwise separation efficiency may be impacted
Immunomagnetic bead separation	Selective isolation of target exosomes	Selection and modification of target-specific antibodies is required, and complex procedure may impact exosome structure and function
Size exclusion chromatography	Simple procedure concurrently isolates exosomes while excluding larger exosomes, protein aggregates, and cell debris	Membrane damage or disruption during isolation may impact exosome function and properties

Early exosome research lack standardization and relies on study-specific protocols. Recently, commercial kits have become more popular, but they often produce exosome preparations of suboptimal purity and quality control [4, 5]. Therefore, establishing standardized guidelines for isolation and benchmarking methods using defined purity and yield metrics would improve compatibility and reproducibility across studies.

Ultracentrifugation is the most widely used exosome isolation technique, applicable to cell culture medium and diverse biofluids such as plasma, saliva, and urine. This method first removes cells and debris via centrifugation, then exosomes are pelleted by high-speed centrifugation. Varying centrifugation speed and duration isolate exosomes of defined size and density ranges. For example, Zhu et al. successfully extracted and identified exosomes from human neural stem cell using ultracentrifugation, which were demonstrated to inhibit the activation of microglia and promote the differentiation of endogenous neural stem cells into neurons [6]. However, a major limitation of ultracentrifugation is vesicle membrane damage and loss during high g-force spinning, which greatly hinders its recovery and application.

Filtration is a method that isolates exosomes from samples containing large particulates. This process involves the passage of these samples through membranes with defined pore sizes. Notably, this technique allows for the isolation of exosomes within specific size ranges. It is particularly effective when applied to cell culture medium and urine, as it can effectively remove non-vesicular debris while allowing exosomes to pass through. A notable application of this technique was demonstrated by Tang et al. [7], who successfully extracted exosomes from HepG2 cells using filtration. They found that these exosomes could deliver chemotherapeutics to cisplatin-resistant cells, thereby conferring drug resistance. However, a significant limitation of filtration is the requirement for multiple centrifugation steps both before and after filtration, which can make the protocol quite cumbersome.

Differential centrifugation separates exosomes based on substance densities. In this process, solutions of decreasing density are layered and then centrifuged at varying forces. This results in the pelleting of exosomes into specific density fractions. A practical application of this technique was demonstrated by Wan et al. [8], who used differential centrifugation to isolate M2 type tumor-associated macrophage (TAM)-derived exosomes. They discovered the intercellular transfer of the MSTRG.292666.16 via extracellular vesicles, which conferred osimertinib resistance in non-small cell lung cancer cells. This revealed a new mechanism of clinical osimertinib resistance in lung cancer. However, one of the challenges of differential centrifugation is the need for precise control over the centrifugation speed and time to ensure proper density-based separation of exosomes.

Based on the decreasing density of the suspension, the density gradient centrifugation method separates exosomes from non-vesicular particles, including proteins, lipids, and RNA aggregates. During centrifugation, the exosome containing components migrate to their equilibrium zone, allowing the isolation of highly purified exosomes from other extracellular material. Using density gradient centrifugation, Tian et al. successfully isolated exosomes derived from irradiated glioblastoma cells and demonstrated that these exosomes could promote tumor growth by increasing FoxP3 expression of differentiating Th1 cells [9]. However, the shortness of this technique is the need for substantial technical expertise to prepare density gradients properly and precise control over centrifugation speed and time to achieve separation. Improper gradient preparation or centrifugation conditions can result in failed fractionation of exosomes from contaminating particles.

Immunomagnetic separation is a technique that uses antibody-coated magnetic beads to selectively isolate exosomes displaying specific surface proteins. This method is known for its high specificity and selectivity. For instance, Guo et al. introduced the immunomagnetic separation method as an emerging strategy for rapid isolation and mild release of exosomes from the 293T cell culture supernatant [10], Arnau et al. isolated exosomes using this method and detected overexpressed exosomal GAPDH transcripts [11]. However, the major limitation of immunomagnetic separation is the need for extensive antibody screening and modification, which adds a significant level of complexity to the method. Additionally, this technique can alter vesicle structure and function due to antibody binding.

Size exclusion chromatography is a method that separates exosomes based on their sizes. In this process, larger exosomes are excluded from alternative-column pores and elute earlier, while smaller proteins and aggregates enter the pores and elute later. Dhananjie et al. conducted a comparison between the precipitation and size exclusion chromatography methods in terms of exosome yields and purity [12]. They discovered that a combination of precipitation followed by size exclusion chromatography resulted in high exosome yields with good purity. On the other hand, Bai et al. tested different size exclusion chromatography-based tandem strategies for plasma exosome enrichment through proteomic analysis [13]. Their findings indicated that the size exclusion chromatography-based tandem methods were superior to the direct size exclusion chromatography method in terms of the purity of exosome isolation from human plasma. However, this technique has its limitations. One major concern is the potential disruption of exosome membranes during separation, which can impair their biological activities. Additionally, size exclusion chromatography is both time-consuming and costly. These factors must be taken into consideration when using this method for exosome separation.

### Drug loading into exosomes

**Fig. 2:**
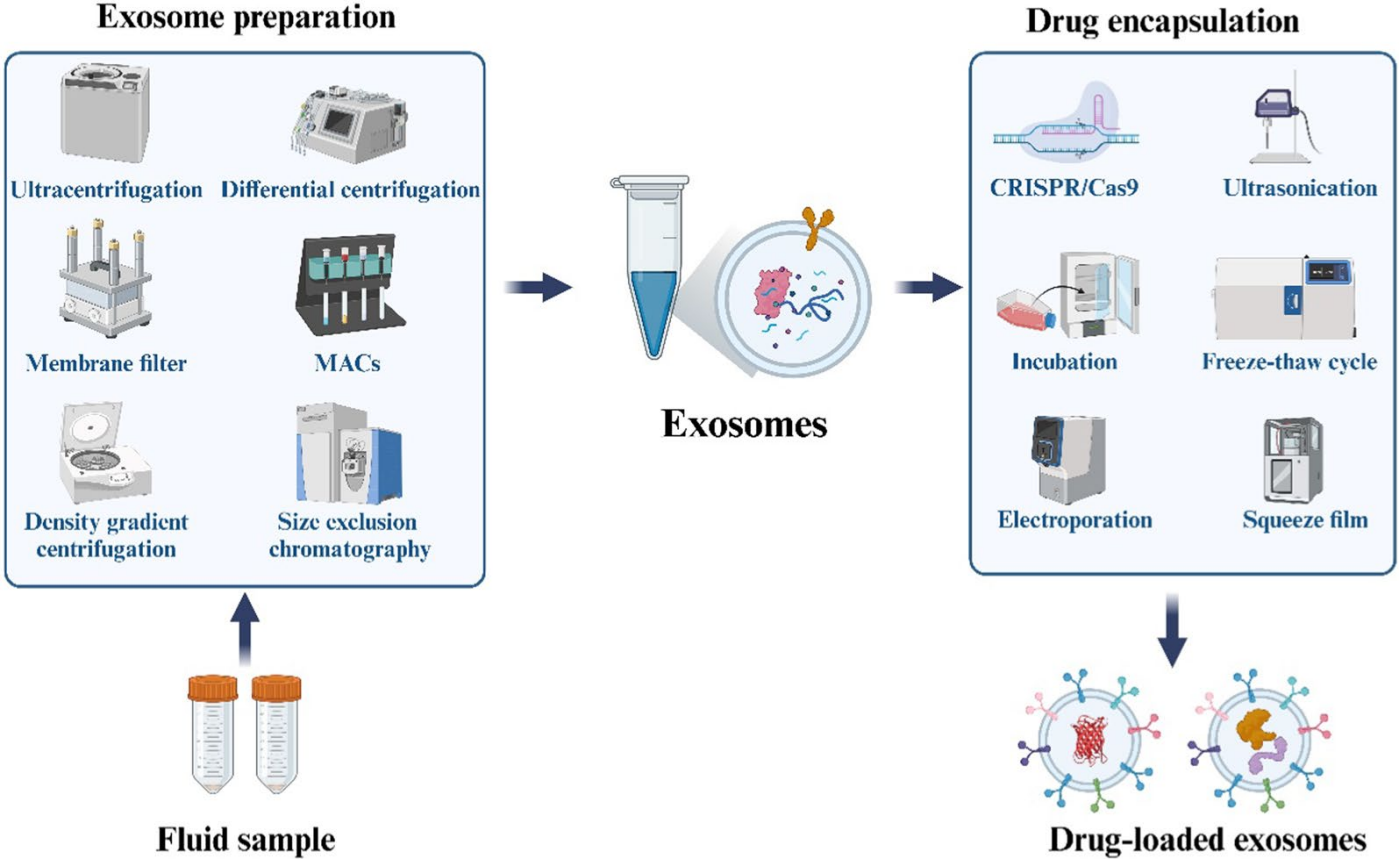
Methods for drug loading into engineered exosomes. Drug loading approaches for exosomes are divided into endogenous and exogenous methods. Endogenous loading primarily utilizes CRISPR/Cas9 technology to package cargo intracellularly. Exogenous loading methods include incubation, electroporation, sonication, freeze–thaw cycles, and extrusion to load cargo after vesicle isolation

Drug loading methods for engineered exosomes can be broadly categorized into two main technical approaches: endogenous loading and exogenous loading (Fig. 2).

#### Endogenous loading method

This method involves transfecting donor cells to express the desired proteins, nucleic acids, or drug molecules using genetic engineering techniques. Subsequently, exosomes containing the bioactive cargo are isolated from the donor cell culture supernatant. An example of this is the CRISPR/Cas9 system for targeted gene editing, which has shown therapeutic promise for cancer [14, 15]. However, its clinical translation has been largely limited by barriers such as immunogenicity. This is where engineered exosomes come into play, as they can overcome these limitations. When exosomes are packaged with the CRISPR/Cas9 system, they directly emerge with the membranes of cancer cells, enabling efficient non-invasive gene editing. A study by Kim et al. demonstrated that CRISPR/Cas9-loaded exosomes could induce apoptosis in ovarian cancer cells by suppressing the expression of poly (ADP-ribose) polymerase-1 [16], which underscores the potential for precision anti-cancer therapy using exosomes engineered with gene editing machinery.

Exosomes are also being investigated as cancer vaccines with advantages over traditional chemotherapies such as higher specificity, controllability, and free of toxicity [17–19]. A recent study demonstrated that, when co-administered with a cancer vaccine, exosomes released by M1-polarized macrophages could boost vaccine immunogenicity and anti-tumor immune responses in mouse models, which shines light on the therapeutic potential of engineered exosomes to develop optimized anti-cancer vaccines [20].

#### Exogenous loading method

This method first isolates exosomes, then incorporates drugs or bioactive cargo externally using various techniques. Compared to the endogenous loading method, the exogenous loading method has several advantages. It offers simpler protocols, greater stability, and improved scalability for large-scale production. Common exogenous loading approaches include co-incubation, electroporation, sonication, freeze–thaw cycles, and extrusion. These techniques provide a versatile and efficient way to load therapeutic agents into exosomes for targeted delivery.

In co-incubation method, exosomes are isolated from cell culture supernatant then incubated with drugs or bioactive molecules. The drug-loaded exosomes are finally purified by ultracentrifugation or filtration. To the greatest extent, this method maintains the integrity of the exosome membranes and is particularly suitable for small hydrophobic molecules. For instance, Zhao et al. loaded docetaxel (DTX) into M1-polarized macrophage-derived exosomes (M1-Exo) using the co-incubation method and they successfully developed a DTX-M1-Exo drug delivery system. Evidenced by in vitro experiments data, they demonstrated that the DTX-M1-Exo could effectively target and kill breast cancer cells, which highlights the potential of the co-incubation method in creating targeted drug delivery systems using exosomes [21].

Electroporation is a technique where an electric field is applied to increase exosome membrane permeability and induce temporary hydrophilic pores, which enables the loading of hydrophilic small molecules like amino acids and peptides. A study by Yan et al. utilized this method to encapsulate miR-31-5p mimics in milk-derived exosomes, which were used as carriers for miR-31-5p delivery. The use of exosomes for miRNA delivery offers several advantages over direct miRNA delivery, such as enhanced cell uptake and resisted enzymatic degradation of miRNAs, as well as increased miRNA bioavailability, which results in improved efficacy for wound healing in diabetics [22]. However, electroporation may cause exosome aggregation and reduce their stability.

Sonication is a method that uses probe sonication to create transient pores in the exosome membrane. This process allows the diffusion of small hydrophilic molecules into the exosomes. Generally, the exosome membrane can be restored after a 1-h incubation at 37 °C post-sonication. Utilizing this method, Gao et al. loaded berberine into M2 macrophage-derived exosomes (Exos-Ber), and the newly generated Exos-Ber was found to reduce the M1 marker iNOS and suppress the expression of M2 marker CD206, which resulted in the promotion of macrophage/microglia polarization from the M1 to the M2 phenotype [23]. In addition, Britta et al. compared the loading capacity to hydrophilic compounds and impact on vesicle functions between different encapsulation methods, they found increased loading capacity in order saponin ≤ sonication < fusion < freeze-thawing ≤ osmotic shock [24]. Notably, it’s important to consider the specific conditions and parameters of sonication to ensure the integrity and functionality of the exosomes.

For the freeze–thaw cycle method, exosomes and drugs are first incubated at room temperature, then undergo 3–5 freeze–thaw cycles between −80 °C and room temperature. After these cycles, the drug-loaded exosomes are purified by ultracentrifugation, but this method can affect the stability of the exosomes and their uptake by cells. Cheng et al. found in HEK 293T exosomes that higher temperature and increased freeze–thaw cycles altered vesicle stability and reduced cell uptake [25]. Therefore, while the freeze–thaw cycle method can be effective for loading drugs into exosomes, careful consideration must be given to the specific conditions to maintain the functionality of the exosomes.

The extrusion method starts by dissolving or suspending the drug to be encapsulated in a solvent such as saline, glycerol or ethanol. The drug solution is then mixed with the exosome suspension, and the mixture passes through a lipid extruder multiple times, which loads the drug into the exosomes. Zhang et al. utilized this method to generate drug-loaded exosomes derived from human umbilical cord MSCs [26]. By conducting proteomic analysis on these exosomes, they identified 1,669 encapsulated proteins. Further experiments demonstrated that these drug-loaded exosomes were able to facilitate wound healing by promoting cell proliferation, migration, and angiogenesis. However, the extrusion method can potentially damage exosomes and impact drug loading efficiency if not properly optimized. Parameters such as extrusion pressure, number of passes, and exosome concentration need to be carefully controlled to avoid disrupting exosome integrity and to achieve efficient drug encapsulation.

## Decoration of exosome for drug delivery and targeted therapy

Exosomes are promising drug delivery vehicles capable of carrying small molecules, proteins, nucleic acids, and other bioactive molecules (Table 2). However, native exosomes exhibit broad biodistribution and lack targeting specificity. Therefore, a major research focus has been developing strategies to decorate the exosomal surface with targeting moieties to direct their delivery to desired cell types and tissues. This section we review recent approaches for functionalization and targeting of engineered exosomes.

**Table 2:** Engineered exosome as delivery vehicles for diverse drug types

Drug types	Drugs	Disease types	In vitro	In vivo	References
RNAs	miR-145-5p	Pancreatic ductal adenocarcinoma	Cancer cell proliferation and invasion↓	Cancerous cells apoptosis↑	[27]
	miR-183-5p	Breast cancer	Growth and metastasis of cancer cells↓	Tumor growth↓	[28]
	miR-1827	Colorectal	Cancer cell proliferation, migration, and invasion↓	Tumor growth and liver metastasis↓	[29]
	Cas9 mRNA	Breast cancer	Cancer cell growth↓	Tumor growth↓	[30]
	piRNA-18	Colon cancer	Cancer cell proliferation↓	Tumor proliferation, migration, and invasion↓	[31]
	circABCB10	Colorectal	Cancer cell growth↓	Tumor growth↓	[32]
Proteins	CIITA	Lymphoma	T cell response↑	T cell response↑	[33]
	NF-κB	Prostate cancer	Androgen receptor inhibition↑	Tumor growth↓	[34]
	TRAIL	Lymphoma	Leukocyte apoptosis↑	No effect on tumor growth	[35]
Compounds	Paclitaxel	Breast cancer	T cell ↑	Tumor growth↓	[36]
	Doxorubicin	Triple-negative breast cancer	Cancer cell growth↓	Tumor growth↓	[37]
	Curcumin	Glioblastoma	Glioblastoma cell growth↓	–	[38]

↑ increase; ↓ decrease

### Targeting ligand display

Display of targeting ligands on the exosome surface enables directed delivery to cells expressing the cognate receptors. Ligands can be attached directly to exosomal membrane proteins or lipids using chemical conjugation or fusion protein approaches. Alternatively, parental cells can be engineered to express ligand-anchored proteins that get incorporated into secreted exosomes.

Peptide ligands typically possess the ability to bind selective receptors or epitopes on target cells. These peptides can be fused to exosomal membrane proteins, a process that equips the exosomes with the ability to target specific cells. As a result, these modified exosomes exhibit enhanced functionality in intercellular communication. For instance, Lamp2b protein was fused with αv integrin-binding iRGD peptide or EGFR-binding GE11 peptide to exosomes displaying these targeting peptides on the surface. The iRGD and GE11 exosomes showed preferential accumulation in αv integrin-positive and EGFR-overexpressing breast cancer cells [39, 40]. Moreover, a cardiac homing peptide (CHP) was anchored onto exosomes via a streptavidin–biotin linkage, and those CHP-displaying exosomes showed enhanced cardiac accumulation after intravenous injection [41, 42], which demonstrated that these peptide ligands could enable functional targeting of exosomes to specific tissues and disease sites.

Surface display of antibody fragments that bind unique cellular epitopes is another common targeting approach. For example, Lamp2b can fuse with an anti-HER2 nanobody and direct exosomes to HER2-overexpressing breast cancer cells, while exosomes displaying anti-EGFR nanobody are found to suppress EGFR signaling and reduce proliferation in triple negative breast cancer cells [43]. Single chain variable fragments (scFv) against tumor antigens have also been anchored using hydrophobic membrane intercalating peptides or enzymatic lipidation, and the scFv-decorated exosomes are found to bind and get internalized by target cancer cells [44]. Furthermore, researchers also generated exosomes displaying scFv against folate receptor, which increased delivery of chemotherapeutic cargo to folate receptor-positive cancer cells [45].

Aptamer ligands are short nucleic acids that bind specific molecules. Previous reports showed that bivalent RNA aptamers against PSMA were conjugated to exosomes using cholesterol anchors, and elevated PSMA-dependent uptake in prostate cancer cells was shown [46]. Also, tetravalent RNA aptamers against EGFR conjugated exosome using click chemistry were proved to enhance targeting in EGFR-overexpressing triple negative breast cancer [47]. Notably, DNA aptamers against nucleolin surface protein were anchored onto exosomes using a biotin-NeutrAvidin connector [48], and this system was proved to be capable of specifically delivering exosomal cargo into leukemia cells in vivo, which, in combination with most recent findings, highlighted aptamers as adaptable targeting ligands to direct exosomes to cancer.

Glycan-binding proteins on immune cells play a crucial role in the removal of exosomes from circulation. One strategy to enhance delivery is to shield the exosome glycocalyx, thereby evading immune clearance and simultaneously displaying targeting glycans. A previous study implemented a metabolic glycoengineering approach to exosomes, which enabled them to present targeting glycans, and the resulted engineered glycoexosomes exhibited lacNAc sugars and demonstrated an affinity for dendritic cells via the DC-SIGN receptor [49]. In a different approach, linear polyglycerol sulfate was applied as a coating to the exosome surfaces, which effectively shielded the immune-activating glycans, while still allowing for the display of targeting mannose moieties [50]. Collectively, this innovative approach significantly increased the potential of exosome to reduce immune elimination while simultaneously enabling directed targeting.

Conjugation of lipids ligands presenting headgroups that engage biological targets provides a direct means for exosomal surface decoration. For example, phospholipids displaying folate groups were inserted into exosome membranes using hydrophobic interactions, and the folate-lipids were found to mediate specific uptake in cancer cells expressing the folate receptor [51]. Several researchers synthetically incorporated glycolipids presenting CD47 "don’t eat me" protein and an αvβ3-integrin targeting peptide, and these glycolipid-exosomes evaded phagocytosis and homed to angiogenic blood vessels [52]. More recently, Gong et al. generated exosomes displaying hyaluronic acid by expressing a membrane anchor protein fused with a hyaluronic acid-binding domain [53], and they demonstrated that the hyaluronic acid-displaying exosomes showed enhanced anti-hepatic fibrosis effect by interacting with CD44 receptors on tumor cells.

### Modulation of endogenous proteins

Exosomal membrane natively contains proteins such as tetraspanins, integrins, and lectins. These proteins can influence the exosome biodistribution through their interactions with target cell receptors. By genetically modulating parental cells to either overexpress or knockdown specific proteins, the targeting capabilities of engineered exosomes can be significantly enhanced.

Tetraspanins comprise a family of scaffolding proteins that mediate vesicle-cell fusion and signaling. Take CD63-rich exosomes as an example, they display tropism towards target cells expressing Tspan8, while knockdown of Tspan8 in mice can reduce exosome uptake in liver and increase accumulation in spleen, brain and heart [54, 55]. Hence, modulating tetraspanin levels can alter exosome biodistribution. Tetraspanins also bind tissue-specific lectins, which support exosomes adhesion to dendritic cells by engaging DC-SIGN receptor, and exosomal CD81 binds hepatocyte asialoglycoprotein receptor through galectin domains [56]. Therefore, strategies to overexpress tetraspanins could therefore enhance targeting.

Integrins are cell adhesion molecules, they play a crucial role in facilitating cell–matrix and cell–cell interactions. Interestingly, exosomes display integrins that determine their capture by recipient cells. For instance, α6β4 and α6β1 integrins present on tumor cell-derived exosomes have been found to bind to lung epithelial cells, thereby promoting metastasis [57, 58]. In addition, the β1 integrin present on exosomes has been shown to influence organotropic metastasis in models of breast and pancreatic cancer. Studies have shown that the knockdown of αv integrin on exosomes reduces liver tropism, while its overexpression increases hepatic localization [59, 60]. Consequently, it is reasonable to conclude that by modulating the levels of exosomal integrins, it is possible to alter their biodistribution profile, which opens up new avenues for improving the targeting capabilities of exosomes, potentially enhancing the efficacy of exosome-based therapies.

Galectins are β-galactoside-binding proteins that can facilitate glycan-dependent interactions between cells and vesicles. One such galectin, galectin-5, when expressed, triggers the secretion of galectin-5-enriched exosomes from Kupffer cells. These exosomes show a preference for hepatocellular carcinoma cells, adhering to their surface glycoproteins [61]. However, when galectin-5 is inhibited, this binding of exosomes is also suppressed, leading to an inhibition of cancer progression. Similarly, the overexpression of another galectin, galectin-9, stimulates the release of galectin-9-expression exosomes from tumor cells, and these exosomes promote angiogenesis [62].

### Targeting motif display

Cell-binding motifs can be displayed on exosome surface using protein engineering approaches, and these approaches commonly include the use of viral targeting motifs, cell-penetrating peptides, and protein transfection domains. Notably, each of these elements plays a unique role in enhancing the ability of exosomes to target and penetrate cells, which makes them valuable tools in the field of targeted drug delivery and disease therapy.

Viral proteins contain short peptide sequences that bind host cell receptors to allow viral entry. Fusing these targeting peptides to exosomal proteins can reroute their tropism. Yu et al. demonstrated this by expressing a peptide from the rabies virus glycoprotein (RVG) on the Lamp2b protein, then this RVG-Lamp2b was displayed on the surfaces of exosomes and was verified to mediate specific delivery of exosomes into neural cells by binding to the acetylcholine receptor [63, 64]. In a similar vein, a peptide from the hepatitis C virus envelope glycoprotein E2 was found to bind hepatocellular carcinoma cells through interaction with the CD81 receptor, and exosomes displaying this H77 peptide showed enhanced accumulation in the liver in vivo [65, 66].

Cationic cell-penetrating peptides (CPPs) like trans-acting activator of transcription (TAT) can translocate lipid membranes through electrostatic interactions and insert into exosomes when fused to proteins [67]. Haney et al. attached TAT peptide to catalase enzyme and loaded them into exosomes for treating Parkinson’s disease [68, 69], where TAT-catalase exosomes were found to display neurotropism and antioxidant effects in the brain.

Certain protein known as protein transfection domains possess an inherent capacity to cross cell membranes. The transgenic membrane permeable (Tango) peptide is one such example, it consists of a membrane translocation sequence derived from the zebrafish voltage-dependent anion channel. When this Tango peptide was fused to the mCherry fluorescent protein and anchored into exosome membranes using a laminin domain, the resulting Tango exosome demonstrated increased cellular internalization compared to unmodified exosomes, indicating that protein transfection domains could potentially enhance exosomal uptake and facilitate the delivery of encapsulated cargo across plasma membranes [70]. However, it's important to note that the tissue targeting ability of these domains may still require further optimization.

## Assessment of drug encapsulation in exosomes

### Assessment criteria

Evaluation of drug loading into exosomes focuses on several aspects, including drug release efficiency, drug stability, drug delivery efficiency, cellular uptake efficiency, therapeutic efficacy, and biosafety evaluation. For Drug release efficiency, it refers to the percentage of drugs released from exosome and indicates the loading and controlled release capabilities. Methods to quantify drug release efficiency include UV–vis spectrophotometry, high performance liquid chromatography (HPLC), fluorescence staining, Tinopal staining, magnetic resonance imaging (MRI), and filtration. Chen et al. developed a graphene oxide-based nanocomposite coated with chitooligosaccharides and polyglutamic acid (GO-CO-PGA) as an exosome-based drug delivery system. Using UV–vis spectrophotometry, they confirmed sustained drug loading efficacy by 73% [71]. Similarly, Sun et al. loaded curcumin into exosomes and examined its efficacy using HPLC [72]. Değirmenci et al. electroporated exosomes derived from normal epithelial breast cells with lapatinib and verified the drug loading efficacy using HPLC, they found that compared to free drug, the lapatinib-loaded exosomes showed higher anti-proliferative effects and enhanced apoptosis induction ability in breast cancer cells [73].

Drug stability describes the ability of a loaded drug to maintain its molecular structure, activity, physicochemical properties, and biological function under certain conditions without being influenced by external factors over time. Methods to assess drug stability include high performance liquid chromatography-mass spectrometry (HPLC–MS), flow cytometry, electron microscopy, and molecular dynamic simulations. Sharma et al. used an immunoinformatic approach to design a multi-epitope tuberculosis vaccine from immunogenic exosomal proteins, and they demonstrated that the vaccine elicited cellular and humoral immunity and provided broad population coverage by compensating for genetic variations. In particular, molecular dynamics simulations were used to model, refine, and dock the vaccine structure to the TLR4 immune receptor [74].

The efficiency of drug delivery is gauged by the quantity of drug that exosomes successfully transport to target cells within a specified timeframe, which is influenced by several factors, including the properties of the exosome suspension, the concentration of the drug, its stability, and the target organ. Various methods are employed to assess drug delivery efficiency, such as fluorescent labeling, biofluorescence imaging, fractionation, transmission electron microscopy (TEM), and scanning electron microscopy (SEM). In a study aimed at promoting cartilage regeneration, Lee et al. utilized the freeze–thaw cycle method to load exosomes with miR-140 [75]. They found that these miR-140-loaded exosomes induced membrane fusion and released miRNA into the cytoplasm. Furthermore, TEM was used to confirm the morphology of the exosomes and the delivery efficiency of miR-140. On a related note, Zhu et al. engineered tumor-exocytosis exosome/AIE luminogenic hybrid nanovesicles for use in photodynamic therapy [76]. They used electroporation to load the photosensitizer DCPy into exosomes. The quantity of drugs packaged into the exosomes was then confirmed using fluorescent labeling.

Cellular uptake efficiency defines the efficacy of exosome drug absorption and utilization by target cells after uptake. It depends on various factors, such as drug properties, exosome loading capacity, exosome/cell membrane permeability, receptor affinity, and metabolic capacity. To assess cellular uptake efficiency, different methods can be used, such as fluorescent/radioisotope labeling, confocal microscopy, and cytological staining. For example, Zhu et al. developed an intraocular lens (IOL) surface modified with exosomes derived from lens epithelial cells (LECs) and loaded with doxorubicin (Dox), an anti-proliferative drug [77]. They used confocal microscopy to demonstrate that the exosome-functionalized IOL enhanced the cellular uptake of Dox by LECs due to the homologous targeting feature of exosome, which resulted in superior anti-proliferation effect and effective posterior capsular opacification (PCO) prevention. A recent development in the evaluation of exosome cellular uptake efficiency is the creation of fluorine-engineered exosomes (exo@FPG3), which are generated through surface engineering of exosomes with fluorinated peptide dendrimers (FPG3), and they are proved to enhance the intracellular delivery and biological activity of exosomes [78]. The study found that the intracellular uptake of exo@FPG3 was energy-dependent and clathrin-mediated endocytosis was a key pathway for exo@FPG3 to enter HUVECs. Moreover, Yang et al. investigated the cellular uptake efficiency of curcumae rhizoma exosome-like nanoparticles (CELNs) loaded with astragalus components (AC) in Caco-2 cell models, and they found that AC-CELNs exhibited superior uptake and transmembrane transport capacity compared to free AC [79].

Therapeutic efficacy assessment involves laboratory assessment of disease status in experimental subjects treated with drug-loaded exosomes to determine if the expected therapeutic outcomes are achieved, it is crucial for establishing the scientific merit, rationale, and safety of a treatment regimen. Better therapeutic efficacy indicates more potent disease treatment by the drug-loaded exosomes. Methods for evaluating therapeutic efficacy include cell viability assays, molecular profiling, animal studies, and clinical trials. Since MSCs have low retention and survival in infarcted hearts, Huang et al. investigated whether exosomes derived from MSCs could enhance treatment of acute myocardial infarction. They found that intramyocardial delivery of exosomes followed by intravenous MSC infusion significantly improved cardiac function, reduced infarct size, and increased neovascularization compared to exosome alone or MSC monotherapy [80]. Xie et al. presented a novel technique for label-free detection of cellular HER2 using machine learning-driven SERS, and they applied their method to dynamically monitor the therapeutic efficacy of drug-loaded exosomes targeting HER2+ breast cancer cells [81]. They showed that their method could capture the variations in HER2 expression and cell viability during the treatment, which could facilitate the therapeutic decision-making and management of breast cancer. Sana et al. investigated the use of exosomes as a natural delivery platform for bleomycin, where they prepared exosomes loaded with bleomycin (Exo-BLM) from cancer cells and tested their effects on tumor cells in vitro and in vivo [82]. Their findings showed that Exo-BLM had high cancer targeting ability and enhanced antitumor activity, and reduced toxicity compared to free bleomycin in a mouse model. Similarly, using a non-invasive liquid-biopsy-based assay, Ting et al. developed a novel approach for assessing the therapeutic efficacy of neoadjuvant chemotherapy (NACT) in patients with advanced gastric cancer (AGC), which was demonstrated to potentially facilitate precision treatment of NACT for patients with AGC [83].

Since exosomes are recognized as promising drug delivery vehicles regarding their ability to traverse biological barriers, a comprehensive biosafety evaluation is imperative prior to clinical translation. This rigorous assessment should encompass several areas: (1) In vitro cytotoxicity and functional assays can determine potential toxicity and adverse effects of exosomes on cells; (2) animal models enable evaluation of in vivo systemic toxicity, immunogenicity, and other safety risks; (3) biodistribution studies are critical for tracking exosome accumulation and clearance kinetics in organs and tissues following administration; (4) pharmacokinetic and pharmacodynamic analyses elucidate exosome stability, metabolism, elimination, and delivery of drug cargo to target sites; and (5) drug interaction studies reveal impacts of exosomes on the safety and efficacy of concomitant medications. For example, Kim et al. evaluated exosomes loaded with the chemotherapy drug paclitaxel and demonstrated that the drug-loaded exosomes are free of cytotoxicity or immunogenicity, but can accumulate in and deliver drug to tumor sites [84]. Jiang et al. loaded exosomes engineered to express TRAIL with the drug triptolide as targeted melanoma therapy. In vitro, the TRAIL-expressing exosomes enhanced tumor cell uptake, inhibited cancer cell proliferation, invasion, and migration, and induced apoptosis. In vivo, the TRAIL-expressing exosomes suppressed tumor progression and reduced triptolide toxicity with ideal biosafety [85].

### Evaluation methods

#### Laboratory testing

TEM enables direct visualization of exosomes such as their morphology, size distribution, cargo abundance, and other physical characteristics. Zhu et al. imaged triple-negative breast cancer cell-derived exosomes by TEM and found proteins involved in extracellular matrix interactions and metastasis [86]. Also, Yu et al. visualized milk-derived and drug-loaded exosomes by TEM, they showed superior osteogenic potential of exosomes encapsulating icariin both in vitro and in vivo [87]. Liu et al. engineered MRI-trackable exosomes by expressing a ferritin-lactadherin fusion protein in parent cells, they demonstrated that the genetic modification had no effect on exosome morphology using TEM, and further MR imaging enabled in vitro tracking and in vivo monitoring of exosomes [88].

To detect drugs encapsulated in exosomes, researchers commonly employ UV–vis spectroscopy. Tanziela et al. (2022) reported a novel drug delivery platform based on exosomes isolated from glioblastoma cells (U87), where they detected the UV–vis absorption and fluorescence spectra from prepared solution of AgNCs [89]. Additionally, Zheng et al. confirmed the assembly of gold nanorods and aptamers on exosomes for targeted cancer photothermal therapy using UV–vis spectroscopy, among which the UV–vis characterization enabled investigation of the photothermal properties of modified exosome [90].

Protein quantification method can rapidly detect the total protein content loaded in exosomes. Although these methods do not provide information on specific protein cargoes, they allow quick measurement of the overall protein loading. By quantifying total protein, these methods enable fast detection of the presence of protein-based drugs in exosomes. Haney et al. studied exosome delivery of the antioxidant enzyme catalase as a potential therapy for Parkinson’s disease. They first isolated exosomes from macrophages using differential centrifugation. To load the exosomes with catalase, they tested passive incubation, freeze–thaw, sonication, and extrusion methods. Western blot analysis showed that sonication and extrusion loading resulted in higher levels of catalase protein in the exosomes compared to passive incubation [91].

Prior to loading into exosomes, the drug can be fluorescently labeled, then fluorescence microscopy was employed to visualize the intensity within exosomes and further determine the successful loading and quantify/visualize cargo distribution. This method is applicable to fluorescently taggable drugs but costly, and it can also visualize interactions between labeled exosomes and cells to elucidate delivery mechanisms. For example, He et al. used fluorescence microscopy to visualize the uptake of PKH26-labeled exosomes by chondrocytes in vitro [69], they found that the injected labeled exosomes accumulated in the joint and attenuated cartilage damage and pain versus controls, and these exosomes could increase collagen II synthesis and improve pain thresholds over 6 weeks.

**Fig. 3:**
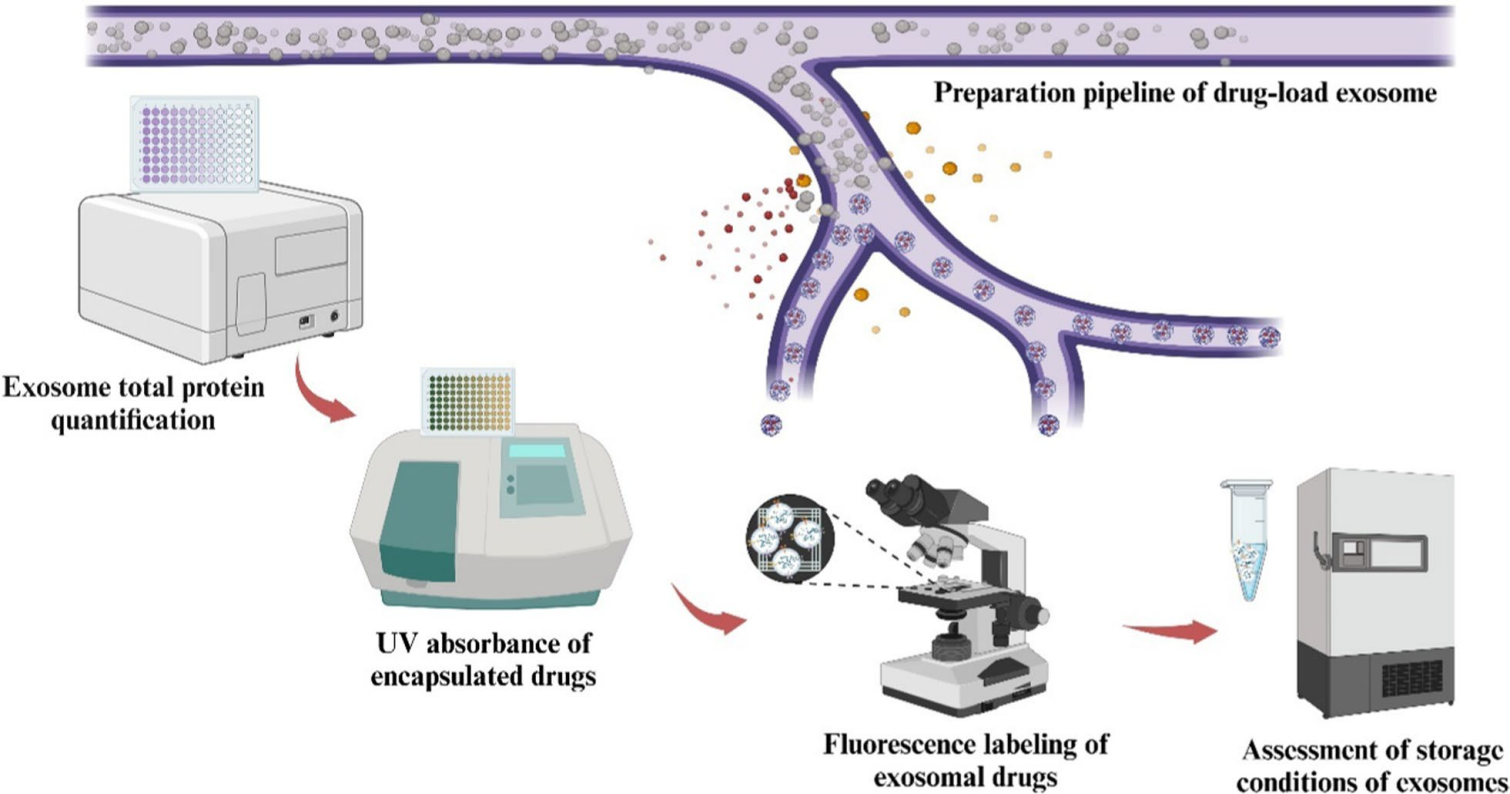
Laboratory detection and evaluation of engineered exosomes. After harvesting drug-loaded exosomes, the total protein is first quantified to determine exosome yield. Next, UV absorbance of the exosome suspension is measured to quantify drug loading efficacy. The engineered exosomes are then fluorescently labeled, enabling evaluation of their biodistribution and drug release capabilities in vitro and in vivo. Finally, storage conditions are optimized by studying the engineered exosomes after freezing for various periods

Stability testing evaluates exosome stability and drug release kinetics by measuring the drug release over time, thereby this method can confirm the drug loading, stability, and guides storage recommendations for drug-loaded exosomes. In area of breast cancer treatment, Moumita et al. performed stability testing of docetaxel-loaded exosomes by measuring the release kinetics, particle size, zeta-potential, and encapsulation efficiency. As a result, they demonstrated promising anticancer efficacy of docetaxel-loaded exosomes against 4T1 breast cancer cells [92]. Other studies found exosomes protected cargoes from enzymatic degradation in blood and from environmental stressors like high temperature and low pH [93–96]. Furthermore, exosomes were found to have the ability in improving drug pharmacokinetics through increased circulation half-life, and these exosomes could be maintained for 24 h at 37 °C (Table 3 and Fig. 3)[97].

**Table 3:** Summary of methods for drug loading analysis in engineered exosomes

Evaluation methods	Advantages	Disadvantages
Morphological observation	Directly observing exosome morphology, size, distribution, and drug loading	Requiring specialized equipment and expertise; sample preparation can be complex, with possible artifacts introduced during processing that may affect sample integrity
UV–visible spectroscopy	Simple, rapid technique to directly quantify contents in exosome samples	Only measuring absorption spectra, does not directly quantify drug loading into exosomes
Protein quantification	Simple, rapid, direct measurements of protein in exosome samples	Only measuring total protein content, does not directly quantify drug loading into exosomes
Fluorescence microscopy	Directly observation of drug encapsulation and quantify exosome uptake	Complex, requiring specialized equipment, extensive sample preparation, and optimization, and potential of introducing artifacts
Stability testing	Assessing stability of exosomes under stressed conditions, providing parameters on exosome physical, chemical, and biological stability	Time-consuming and require stringent storage conditions

#### Cell experiments

**Fig. 4:**
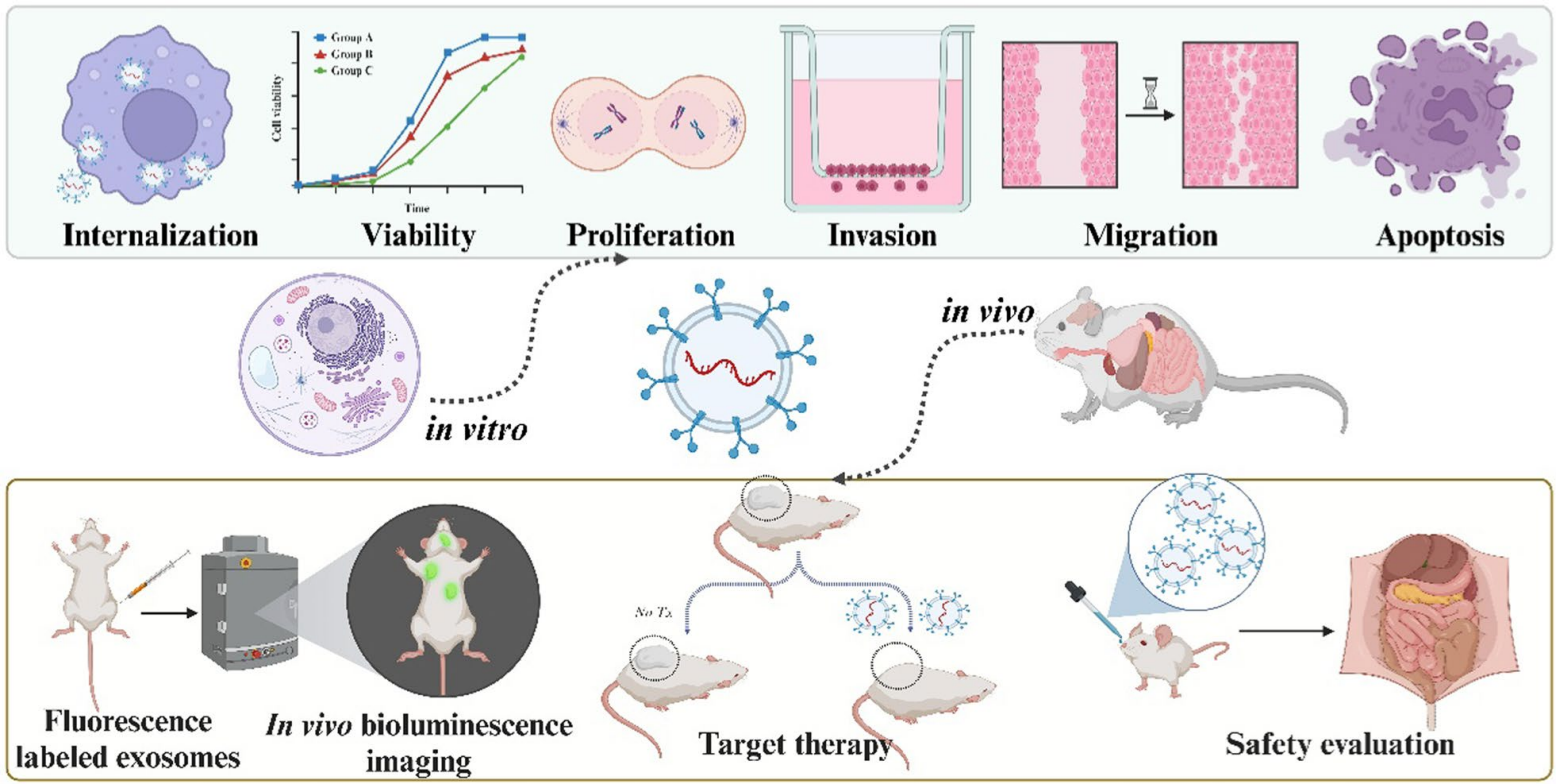
Cellular experiments and animal models for testing engineered exosomes. In vitro cell studies assess exosome-mediated drug delivery by quantifying drug abundance within cells, measuring changes in cell viability or functionality after exosome treatment. In vivo animal studies establish disease models in rodents or other organisms to test exosome biodistribution and therapeutic efficacy

Cell experiments are fundamental for validating exosome drug loading capacity and assessing exosome pharmacological activity (Fig. 4). Methodology, drug-loaded exosomes are added to cultured cells, then the changes in drug levels, cell viability, function, and other endpoints are quantified to evaluate the drug delivery efficacy, which enables the investigation of drug affinity, pharmacology, and mechanisms. For instance, drug-loaded exosomes had shown promise for cancer therapy, and available studies had demonstrated that they could modulate cell viability, apoptosis, migration, proliferation, invasion, angiogenesis, metastasis, and drug resistance across cancer types [98–102]. However, as factors like cell type, density, timing, and detection methods can influence the evaluation results, standardizing assays improves reliability are urgently needed. For instance, immunomodulatory exosomes, such as those loaded with STING agonists or IL-12, can activate anti-tumor immunity and are currently in clinical trial [103], while exosomes delivering KRAS-G12D siRNA can also silence this oncogenic mutation and reduce mutant cancer cell growth []. Hopefully, exosomes loaded with paclitaxel or Dox were demonstrated to overcome drug resistance and improve cytotoxicity compared to free drugs.

#### Animal experiments

Animal experiments are crucial for evaluating drug-loaded exosomes before human testing, as preclinical studies enable assessment of pharmacokinetics, dynamics, and toxicity as required by regulations. Animal models, such as mice and rats, possess similar physiologies to humans and are generally cost-effective and easily managed. Usually, researchers administer drug-loaded exosomes into these testing animals, then they monitor the biodistribution of the drug-loaded exosomes and observe the effects on the animals, including physiological and behavioral changes as well as histological effects [104–107]. The use of small animal models thus allows for controlled evaluation of drug-loaded exosomes before advancing to human testing. For instance, exosomes loaded with chemotherapy drugs inhibited tumor growth in colon and glioblastoma cancer models, and those exosomes were found in tumors with enhanced efficacy over free drug [108, 109]. Moreover, using cardiovascular disease models, Cheng et al. revealed accumulation of angiogenic drug-loaded exosomes in heart tissue, which was demonstrated to promote vessel growth [110].

## Clinical applications

### Cancer

**Fig. 5:**
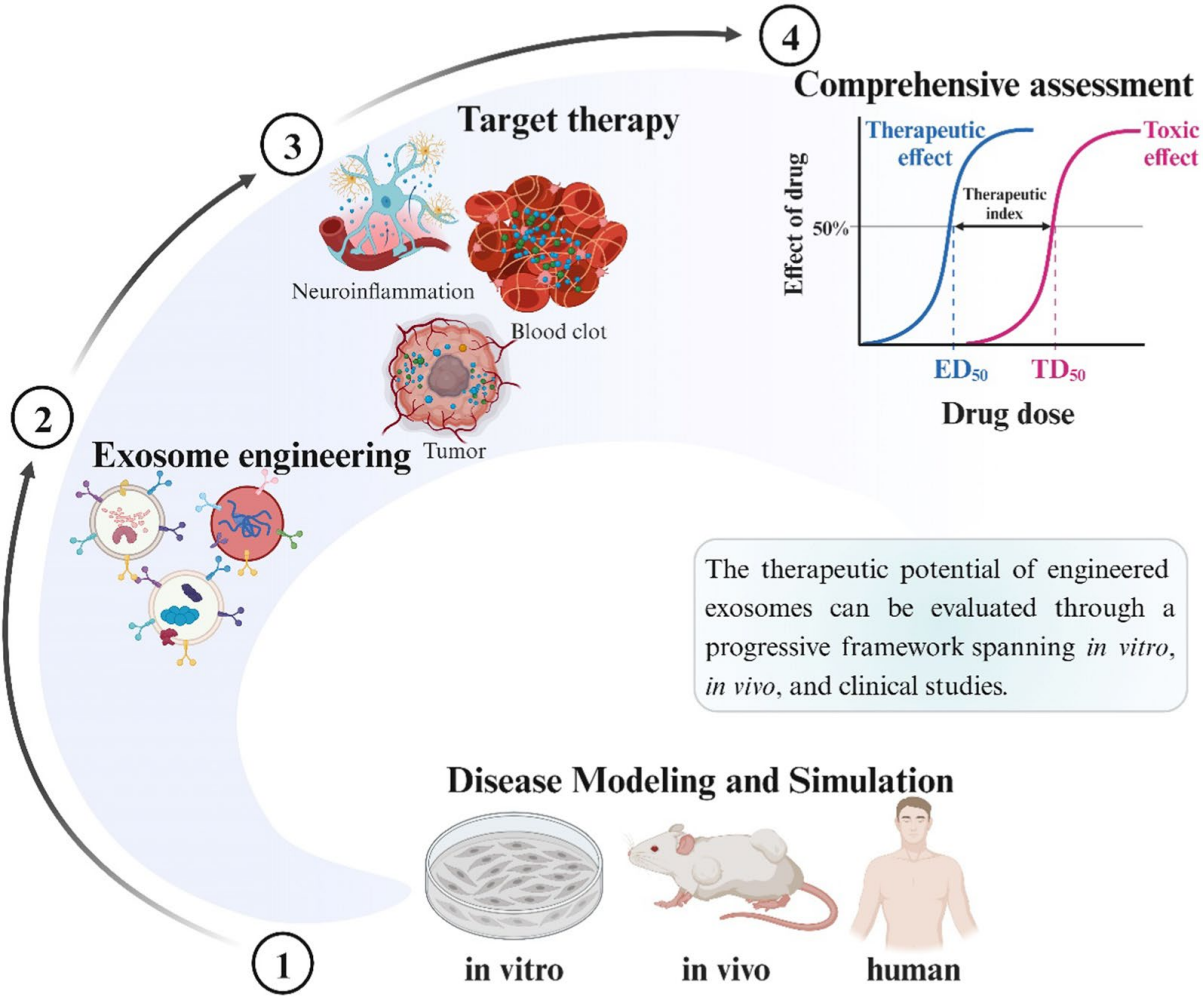
Schematic diagram showing the therapeutic evaluation of engineered exosomes through a progressive framework spanning in vitro, in vivo, and clinical studies

The therapeutic potential of drug-loaded exosomes for cancer treatment can be evaluated through a progressive framework spanning in vitro, in vivo, and clinical studies (Fig. 5). Initial characterization involves assessment of exosome properties such as size, morphology, surface markers, and drug encapsulation and release. In vitro cancer cell studies focus on cellular uptake of exosomes, cytotoxicity, and anti-proliferation effects. While in vivo animal models enable examination of the biodistribution, pharmacokinetics, tumor accumulation, and anti-tumor efficacy of the drug-loaded exosomes. Additionally, the toxicity of exosome-loaded drugs is also monitored through changes in body weight, blood counts, biochemistry, and histopathology [82, 104, 111].

Early-stage human trials primarily concentrate on the safety, maximum tolerated dose, pharmacokinetics, and preliminary efficacy of drug-loaded exosomes [112]. Larger randomized controlled trials are then needed to firmly establish clinical safety and efficacy parameters. The evaluation process is methodically structured, beginning with basic lab studies that assess formulation and biological interactions, followed by preclinical animal models that examine tumor-targeting and efficacy. The final step involves phased human trials that thoroughly assess clinical safety and therapeutic potential. This stepped evaluation provides a comprehensive characterization of drug-loaded exosome formulations as cancer nanomedicines.

In two review article by Xu et al. and Lee et al., they both indicated that various techniques could effectively load chemotherapeutic drugs into exosomes, with electroporation and membrane permeabilization approaches yielding high encapsulation efficiency. In vitro studies demonstrated enhanced cellular uptake, cytotoxicity and anti-proliferation effects of drug-loaded exosomes across diverse cancer cell lines, while animal models revealed superior pharmacokinetics, including extended circulation, tumor localization and anti-tumor efficacy compared to free drug or nanoparticle formulations [113, 114]. However, some toxicity issues like altered blood cell counts and inflammation have been noted and require careful evaluation. Early phase human trials display feasibility and preliminary signs of safety and efficacy, but significant clinical development on drug-loaded exosomes is still needed.

### Neurological diseases

In line with the application of exosomes in cancer treatment, a systematic framework has also been developed for treating nervous system diseases [115–117]. This process begins with the biophysical and biochemical characterization of exosomes, which includes assessing the exosome size, morphology, cargo loading, and interactions with nervous system cells. Subsequent animal studies are conducted to examine the biodistribution of these exosomes, their ability to cross the blood–brain barrier, target engagement, therapeutic effects, and toxicity parameters. The next phase involves early human trials that focus on feasibility, maximum tolerated dose, pharmacokinetics, and preliminary proof-of-concept for safety and efficacy. Following this, larger controlled studies are carried out to rigorously evaluate the clinical effectiveness of exosomes on relevant neurological outcome measures. Throughout these assessment procedures, multifaceted techniques tailored to each stage are employed to systematically validate stable and efficient drug encapsulation, nervous system delivery, therapeutic effects, and clinical safety.

Given the prevalence of neurological diseases like Alzheimer’s and Parkinson’s and the current lack of effective treatments, exosomes have shown promising therapeutic effects in both in vitro and in vivo models of neurodegenerative diseases, which positioned them as a novel strategy for neural repair. Recent studies have revealed that those drug-loaded exosomes can promote neural growth and regeneration by releasing bioactive molecules like neurotrophic factors and miRNAs. Besides, some exosomes can also target neural cells by modifying their surface proteins to enhance treatment effects [118]. Collectively, the potential of exosomes in the treatment of neurological diseases is promising, and ongoing research continues to explore their full therapeutic potential.

### Cardiovascular diseases

Exosomes are emerging as therapeutic strategy for delivering drugs and treating various cardiovascular diseases. By engineering exosomes to carry targeted drug payloads, researchers can leverage the innate biology of exosomes to deliver therapies to specific sites in the body. Over the past years, there has been significant progress in developing exosome-based therapies for major cardiovascular diseases. Take myocardial infarction as an example, it is commonly known as a heart attack and results from blockage of blood flow to the heart muscle leading to cell death. Several studies have shown promise for exosome therapy to reduce damage and improve healing after myocardial infarction. For example, stem cell-derived exosomes containing specific miRNAs and circRNAs were found to reduce apoptosis and increase proliferation of cardiomyocytes in AC16 cell and mouse models of myocardial infarction [119, 120]. Additionally, exosomes have been engineered to deliver pro-angiogenic factors to stimulate new blood vessel growth after infarction. In 2022, Hu et al. prepared exosomes carrying islet-1 (ISL1) which improved the survival and angiogenesis of endothelial cells and accelerated the recovery of myocardial infarction in a mouse model [121]. Similar with myocardial infarction, emerging progresses were also reported in pulmonary hypertension, atherosclerosis, and heart failure. One study loaded exosomes with miR-211 and found they could contributes to pulmonary hypertension via attenuating CaMK1/PPAR-γ axis in rats [122]. In other work, Li et al. engineered exosomes with platelet membrane phenotype to inherit the targeting ability towards injured endothelial cells which were proved to be able to regress atherosclerosis in mice [123]. While still an emerging field, additional work is needed to scale up manufacturing and evaluate clinical safety and efficacy through human trials. With continued research, exosome nanotherapies could provide transformative new paradigms in cardiovascular treatment.

### Immune system diseases

Immune disorders are well defined as abnormal self-attack of normal tissues due to immune system dysfunction, and diseases related to immune disorders include rheumatoid arthritis, lupus, myasthenia gravis, and multiple sclerosis. Casually, these diseases are associated with varying degrees of tissue/organ damage [124]. For instance, You et al. engineered stem cell-derived exosomes to display molecules targeting macrophage reprogramming in rheumatoid arthritis, using flow cytometry and immunofluorescence microscopy, they tracked in vitro and in vivo the transportation of exosomes. The in vivo stability monitoring demonstrated that exosomes could precisely regulate macrophage activity and functionality [97]. Additionally, Wu et al. employed exosomes to deliver bryostatin-1, and they reported that the bryostatin-1-loaded exosomes could promote remyelination and neuroprotection in a cuprizone-induced demyelination model of multiple sclerosis. Hopefully, the bryostatin-1-loaded exosomes were found to effectively protect neurons and stimulate remyelination as a novel treatment strategy [125]. Fang et al. engineered exosomes to display CD40 receptor, and they showed the ability of exosomes in modulating B cell activation and demonstrated it could alleviate systemic lupus erythematosus nephritis [126].

## Conclusions and perspectives

### Conclusions

Exosomes have emerged as promising biological nanoparticles for targeted drug delivery due to their biocompatibility, stability, and ability to encapsulate diverse cargoes. However, standardized methods are still lacking for the fabrication, optimization, and analysis of drug-loaded exosomes. This review highlights current techniques to address these gaps, including: (1) endogenous and exogenous loading methods that enable encapsulation of cargoes into exosomes; (2) characterization criteria such as quantification of drug loading, stability, release kinetics, cell uptake, biodistribution, pharmacokinetics, and therapeutic outcomes in preclinical models to evaluate exosomes as delivery vectors; and (3) advances in isolation, modification, cargo loading, and rigorous lab and animal testing that are paving the way for clinical translation of exosomes.

In summary, the current review synthesizes cutting-edge bioengineering strategies enabling the production of exosomes with enhanced drug delivery capabilities, and the establishment of robust protocols for manufacturing, optimization, and analysis will accelerate the development of exosomes as next-generation therapeutics. In the future, continued progress in this emerging field would firmly hold great promise in leveraging exosomes as targeted, multifunctional nanocarriers to address unmet clinical needs across diverse disease contexts.

### Future perspectives

The exciting potential of exosomes has garnered tremendous interest, but realizing this potential in the clinic remains a challenge. There are several critical priorities that need to be addressed to advance exosomes towards clinical translation. These include:

- Scalable production methods must be developed to enable manufacturing of quality-controlled exosomes at therapeutic quantities using bioreactors and defined cell sources;
- Drug loading techniques should be optimized to maximize stable encapsulation of bioactive cargoes via endogenous and exogenous methods, improving delivery capacity;
- Mechanistic understanding is needed of how exosomes traverse barriers, interact with targets, and deliver cargo intracellularly after uptake;
- Versatile isolation and characterization methods require validation and standardization for reproducibility across research groups;
- Rigorous preclinical biosafety assessments are imperative to evaluate risks prior to human trials;
- Pilot clinical studies should demonstrate feasibility, biodistribution, and preliminary efficacy before scaling up.

The realization of exosomes for clinical nanomedicine will require continued cross-disciplinary collaboration between bioengineers, pharmacologists, and clinicians. Archiving the above goals through systematic applied research will unleash the full potential of exosomes to address diverse unmet medical needs.

## Data Availability

Not applicable.

## Abbreviations

TAM: Tumor-associated macrophage
AC: Astragalus components
AGC: Advanced gastric cancer
CELNs: Curcumae rhizoma exosome-like nanoparticles
CHP: Cardiac homing peptide
CPPs: Cell-penetrating peptides
Dox: Doxorubicin
DTX: Docetaxel
exo@FPG3: Fluorine-engineered exosomes
Exo-BLM: Exosomes loaded with bleomycin
Exos-Ber: Berberine into M2 macrophage-derived exosomes
FPG3: Fluorinated peptide dendrimers
HPLC: High performance liquid chromatography
HPLC–MS: High performance liquid chromatography-mass spectrometry
IOL: Intraocular lens
ISL1: Islet-1
LECs: Lens epithelial cells
M1-Exo: M1-polarized macrophage-derived exosomes
MRI: Magnetic resonance imaging
NACT: Neoadjuvant chemotherapy
PCO: Posterior capsular opacification
RVG: Rabies virus glycoprotein
scFv: Single chain variable fragments
SEM: Scanning electron microscopy
TAT: Trans-acting activator of transcription
TEM: Transmission electron microscopy
